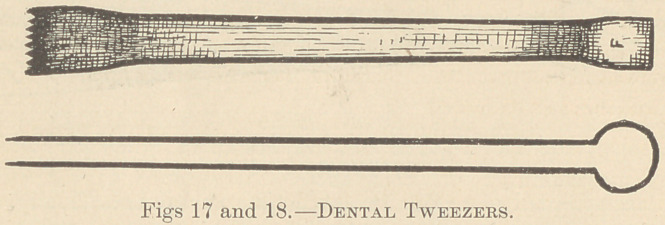# Current News and Opinion

**Published:** 1888-01

**Authors:** 


### Current News and Opinion.

*Editor Independent Practitioner:*

While visiting a Museum at Naples, I happened to see a set of dental instruments which were found in Pompeii, and I was led by curiosity to examine and sketch several of them in my note-book. There were four varieties, and several sizes of each variety. A copy of these sketches, with one specimen of each, is shown in the six following figures. These instruments consisted of forceps and tweezers of different sizes and patterns. They appeared to have been intended as aids in the use of wire or strings for binding in line loose teeth, so that they would be firmer. They were also peculiarly adapted for placing strings around teeth for the purpose of extraction.

Fig. 13.—Dental Forceps Found in Pompeii.

The largest instrument (Fig. 13) was a pair of flat transversely serrated beaked forceps, about eight inches in length, which, though smaller, resembled those now used by shoemakers for drawing the "uppers" over the bottom of their lasts. The beaks of this instrument, which were evidently made of bronze, were about one-fourth of an inch in width.

Fig. 14.—Saw-Beaked Forceps.

Fig. 14 illustrates another variety of forceps, an instrument about five inches in length. In the beaks were longitudinally fixed blades, having teeth which locked when brought together, fitting into each other like those in the jaws of a steel trap. Near the end of each saw-blade was a hole, evidently made for a string or wire for adjustment about the teeth.

Figs. 15 and 16.—Dental Tweezers.

Figs. 15 and 16 illustrate different views of one of a variety of tweezers, evidently made of bronze, which were probably intended for adjusting strings or wire. These instruments resembled in form the tongs used by blacksmiths, but they were as thin as the handle of a silver teaspoon; they were from a quarter to a half inch wide, and from three to five inches in length, and about one-sixteenth of an inch or less in thickness, the edges being smooth. The following figures, 17 and 18, illustrate still another variety of tweezers, apparently made for the purpose of passing wire or string between roots.

Figs 17 and 18.—Dental Tweezers.

From all appearances, teeth were generally extracted at this period with strings adjusted by the tweezers and drawn by means of larger forceps (Fig. 13), or by winding the string about the hand. Although I have shown but one sample of each, there were several instruments of each kind in these collections, varying in size. Respectfully, J. N. Farrar.

---

*Editor Independent Practitioner:*

At the American Dental Society of Europe's fifteenth annual meeting, held at Coblentz, Germany, September, 1887, Dr. Miller, of Berlin, in discussing Dr. Cunningham's paper on education, made use of the following language (published in Independent Practitioner for December): "We have a record of the foreign diplomas improperly granted, and the Pennsylvania school heads the list, with the Philadelphia a good second; Baltimore stands next, and New York is fourth. No other college has issued more than three. The Pennsylvania and Philadelphia colleges have done more harm here by awarding diplomas to unworthy persons than sham institutions have by selling them."

Dr. Miller is a scientific man, and has therefore learned the value of accuracy in speech as well as work, and we infer that he has not in the least deviated from what he would regard as strictly true in making the above statement regarding the American schools. As the faculty are, individually, intensely interested in the Pennsylvania College of Dental Surgery, we should like Dr. Miller to answer the following questions :

1. When he speaks of the "Pennsylvania School," does he allude to the Pennsylvania College of Dental Surgery ? if so,

2. What does he mean when he states that a diploma has been improperly granted ?

3. Will he please state to whom these diplomas were granted by the "Pennsylvania College ?"

4 Will he also state the year in which they were granted and the date of the diploma ?

Dr. Miller says "he has a record;" having a record, these questions can all be easily answered. The Faculty of the Pennsylvania College of Dental Surgery are desirous that its *name* and *fame* shall be as free from blemish as possible, and that it shall, in future, be, as it has endeavored to be in the past, free from "doing harm" to a profession which it has for years been laboring to educate and elevate. We, therefore, ask, in justice to ourselves, and to said college, that a speedy answer be given to these several inquiries.

Criticisms of the above general nature do little or no good, and leave the impression that they have originated in a spirit of jealousy or personal pique, which we shall be sorry to attribute to a man of Dr. Miller's scientific eminence and professional ability. We, therefore, hope he will, without delay, furnish names and dates as requested.

C. N. PEIRCE, DEAN.

---

### DAVID M. PARKER., M. D.

At the 20th annual meeting of the American Academy of Dental Science, held in Boston, Nov. 16, 1887, the committee appointed to submit resolutions concerning the death of Dr. David M. Parker, reported the following, which were unanimously adopted:

*Resolved*, That the American Academy of Dental Science has received with sincere sorrow the intelligence of the decease of our late respected friend and associate, David M. Parker, M. D., of Boston, an honorary member and former President of this society.

*Resolved*, That by the death of Dr. Parker this Academy has lost one of its most worthy members, a man of excellent judgment and skill in his profession, and always interested in all movements looking towards the better organization and education of the profession and the advancement of its practice. He was highly esteemed in the community in which he lived, because of his kindly virtues and his earnest, upright and sincere life.

*Resolved*, That the proceedings of this Academy this day, in honor of our late lamented brother, be engrossed upon the records, and communicated to the widow of the deceased, with the assurance of our deep sympathy in her bereavement. Also that a copy be transmitted to the INDEPENDENT PRACTITIONER, the *Dental Cosmos*, and the *Boston Medical and Surgical Journal* for publication.

ELISHA G. TUCKER,
JACOB L. WILLIAMS, } Committee.
EDWARD N. HARRIS.

### FIRST DISTRICT DENTAL SOCIETY.

The annual meeting will be held in Masonic Temple, Twenty-third Street and Sixth Avenue, New York City, January 16, 17 and 18, 1888.

The last annual meeting of this society was one of the largest and most successful ever held in America. Judging by the programme (for which we have not the necessary space), the meeting for 1888 will be fully as interesting and noteworthy as any of its predecessors. The clinics, which will be held at the New York College of Dentistry, will form a special feature of the meeting, and will occupy the forenoon of each day. A large number of operators have promised to demonstrate their specialties

Any dentist who has not received a direct invitation is requested to consider himself personally addressed by this general notice, and may rest assured of a cordial welcome. Programmes and further particulars may be obtained by applying to the President, Dr. W. W. Walker, 69 West 9th Street, or to Dr. A. L. Northrop, Chairman Executive Committee, 57 West 49th Street, New York City.

---

### FILLING ROOT CANALS.

There are so many ways of filling root canals that I feel like apologizing for suggesting another. I have met with such good success with the use of Beta Naphthol, that a few simple directions may be of service to some one who has not yet found a method that is always satisfactory. Not that any claim of infallibility is to be made for this one, which is not yet old enough, though so far, all roots so filled have done well.

Directions.—Melt the crystals into small lumps, and put one or two of them in the pulp chamber. Then with a suitable shaped instrument, heated sufficiently to melt the naphthol, flow it into the canals, and also allow enough to remain in the pulp chamber above the canals to partly fill this. A piece of gold is then to be placed over the now solid naphthol, and the filling with gold is completed.

W. H. Rollins.

---

Dr. J. H. Gartrell, of Penzance, England, for whom Dr. Younger implanted two lateral incisors at a clinic given during the meeting of the International Congress in Washington, reports through the *Dental Record*, of London, that the teeth were lost within two weeks. He says that they got so loose about ten days after insertion that he could not get on with them, and on the twelfth day a dentist in Ottawa, Canada, replaced them again, when he kept them for another day; then the silk ligature gave out once more, whereupon further efforts to keep them in place were abandoned.

The cases were unfavorable ones. Dr. Younger operated against time, and one of them only consumed six minutes from the commencement to the finish. They were retained only by silk ligatures, which soon slackened and allowed the teeth to become loose. Common surgical prudence would seem to demand that they be held immovable, for no one would expect union of the fractured ends of a bone if movement was permitted.

If there is any one thing that will test the amount of saving grace that a man possesses it is to have dealings with the Post-office Department. For years it has been the custom to place upon the one-cent envelope a request to deliver to some other person a circular that did not find the one to whom it was addressed, and to place the name of the firm or corporation upon it, with an announcement of business. Some post-office sharp has just discovered that this is illegal, and has reversed all the previous decisions of the department without notice, leaving business firms, etc., with perhaps many thousands of such envelopes on their hands and unmailable. Every dumb ass who gets a position of power in the department, finds it incumbent upon himself to do something to let the people know that he is *in* the saddle instead of *under* it, and straightway makes a decision that disturbs the whole course of business and causes great inconvenience and expense to every business man in the country. There is not enough of sound business sense in the whole department at Washington to run a Chinese laundry.

---

The following letter is but one of many such received. We have yet to hear from those who have received the book anything but the highest commendation.

"The copy of 'The Microscopic Structure of a Human Tooth' came to-night. *It is just beautiful.* It should be in every dental office in the land. It may well be called a study, for it is a perfect one. It cannot be examined without profiting the examiner greatly. How you can afford to send it at the price I cannot understand. I owe you many thanks for giving me the opportunity to obtain it, and shall always entertain a feeling of gratitude to the *I. P* , not only for all the good which I have derived from it, but for this crowning instance of its devotion to the wants of dentists."

---

Shearjashub Spooner, whose name is well known to all dentists acquainted with the history of their profession, says in the preface to his "Essay on the Art of the Manufacture of Mineral, Porcelain or Incorruptible Teeth," which was published in 1838, that he had lately made from New York a tour to Philadelphia, Baltimore and Washington, for the purpose of gaining dental information, and that of more than twenty dentists upon whom he called, he found only two *willing to exchange secrets* in the making of teeth.

---

For some months the editor of this journal has been using in practice the "Imperial Alloy," an advertisement of which appears in this number, with most satisfactory results. In color, working qualities and apparent strength, it is among the best of the very many with which he has experimented. So far, it does not seem to shrink in the tooth, or exhibit any tendency to assume a spheroidal shape, while it retains its polished surface to a surprising degree.

---

An Archer chair in good serviceable condition, with footstool, spittoon and operating table complete, may be purchased for twenty-five dollars. Apply to the editor of this journal.

Drs. T. E. Weeks and M. G. Jenison, of Minneapolis, send us, neatly bound, a volume containing some hundreds of cuts of the full human denture, intended for the registration of operations in the mouth, for recording fillings and fcr examination charts. There is an index for the names of patients to facilitate reference, with blanks for necessary registration on each page. The binding of these charts in such a compact and convenient form was a happy thought

Prof. Truman, of the Philadelphia University, suggested the combination of iodoform with arsenious acid for devitalizing exposed dental pulps without causing pain. We recently made a paste of these two agents, rubbed together, and moistened with carbolic acid, and applied it in an aggravating case of pulpitis. The object desired was effectually accomplished, and without giving the slightest degree of pain to the patient. F.

Dr Horatio C. Merriam, of Salem, Mass , has compiled a directory of responsible firms engaged in the manufacture or sale of implements and materials used by dentists, and of which the depots do not keep full supplies. Such things as jewelers' tools, broaches, blow-pipes, sheet metals, draw and screw-plates pliers, pivot wire, benches, etc., etc , may be obtained of the dealers, whose names are mentioned, at first hands.

The January Number of *Scribner's Magazine* opens the second year of its publication. The success of its first year is well known, and its second promises extremely well if we may judge by its prospectus of what is to come. The illustrations have steadily improved, and the publishers promise that during 1888 they will be better than ever. A prospectus will be sent to any one upon application to Charles Scribner's Sons.

R. I. Pearson & Co., of Kansas City, publish the neatest, handiest, most complete pocket appointment book that has come to our notice. It is furnished as a premium to subscribers for the *Western Dental Journal.* That publication really needs no premium to commend it to the profession, and the appointment book needs no *Journal* to further its reception, but both together should be irresistible to western dentists.

Dr. L. D. Caulk, manufacturer of "Caulk's Filling Materials," and publisher of "Caulk's Annual," has removed his office and salesroom to Nos., 1305 and 1307 Arch Street, Philadelphia, Pa., although his laboratory still remains in Camden Del. The Doctor has greatly increased his business facilities, and we are informed that his specialties find a market in every civilized country in the world.

Sugar of milk has the property of rapidly dissolving the calcium deposits between the teeth. It therefore forms a valuable dentifrice.—*Exchange.*

Nitric acid has the same property. Is it therefore a valuable tooth-wash ? People should not talk of what they do not understand. Sugar of milk has no such property as is claimed for it.—Editor.

Dr. E. D. Downs, of Owego, whose leg was amputated at the hip for a disease caused by an injury received from his dental chair, is slowly recovering, although for some time his prospects for life were very poor indeed.

## Figures and Tables

**Fig. 13. f1:**
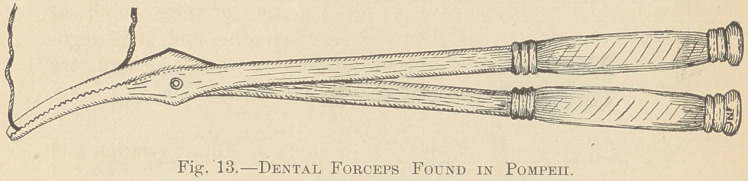


**Fig. 14. f2:**
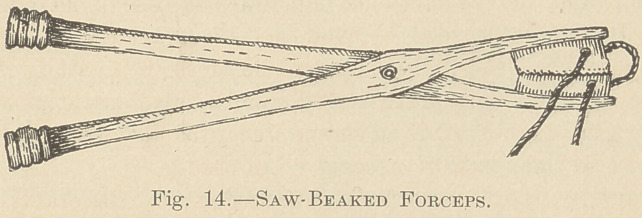


**Figs. 15 and 16. f3:**
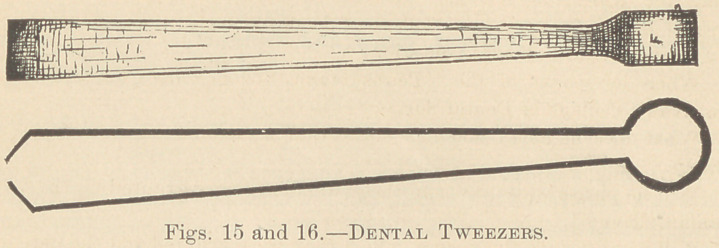


**Figs 17 and 18. f4:**